# Modulating Viscoelasticity and Live Cell Response with Fibrillar Peptide Polymers

**DOI:** 10.1021/acscentsci.6c00960

**Published:** 2026-06-17

**Authors:** Clara Middleton, Laurel Kroo, Todd Emrick

**Affiliations:** Polymer Science & Engineering Department, University of Massachusetts, Conte Center for Polymer Research, Amherst, Massachusetts 01003, United States

## Abstract

Motivated by the sensitivity of cancer to the mechanics of surrounding tissue, synthetic fibrillar peptide hydrogels successfully controlled growth.

## Graphical abstract

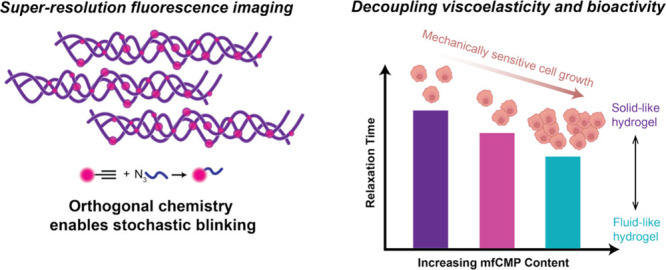

It is often difficult to exactly mimic the immense complexity of biological systems via chemical synthesis and materials assembly. In such cases, the materials community has pursued meaningful reduction of such complex systems to capture essential physicochemical functionality—but with far simplified fabrication and design approaches. Efforts in this field of bioinspired materials research are of immense importance—on one hand, insights gained into structure–property relationships inform next-generation solutions in medicine and therapeutics, while on the other hand, fundamentally new materials systems are created, such that the guidance of biology underpins advanced materials discoveries in areas such as self-repair, actuation, and active matter.

In *Synthetic Surrogates of Collagen-Rich Microenvironments: Integrating Modular Bioactive Fibrillar Structure and Tunable Viscoelasticity via Multifunctional Assembling Peptides*,^1^ Kloxin and co-workers creatively merge crucial impacts of materials design—spanning chemistry, rheology, and biological response—to advance novel synthetic materials used for tissue culture in biomimicking environments pertinent to cancer research. Building on the fundamental importance of soft structures in biology,^2^ the authors successfully replicate essential mechanical features of the extracellular matrix (ECM) in a fully synthetic, abiotic system. Specifically, they prepare hydrogels that are capable of flow and dissipation over biologically relevant time scales by leveraging hydrogen bonding and ‘sticky’ supramolecular interactions within the network. Notably, efforts to utilize synthetic polymers to create bioresembling materials are growing at a rapid pace across numerous fields, including peptide-rich structures,^3^ smart droplets,^4^ and mechanically responsive systems.^5^ Kloxin and co-workers take a novel and comprehensive approach, as illustrated in Figure 1, to this challenging intersection through their preparation of tunable materials designed to study cancer cell proliferation under mechanically realistic conditions.

**Figure 1:**
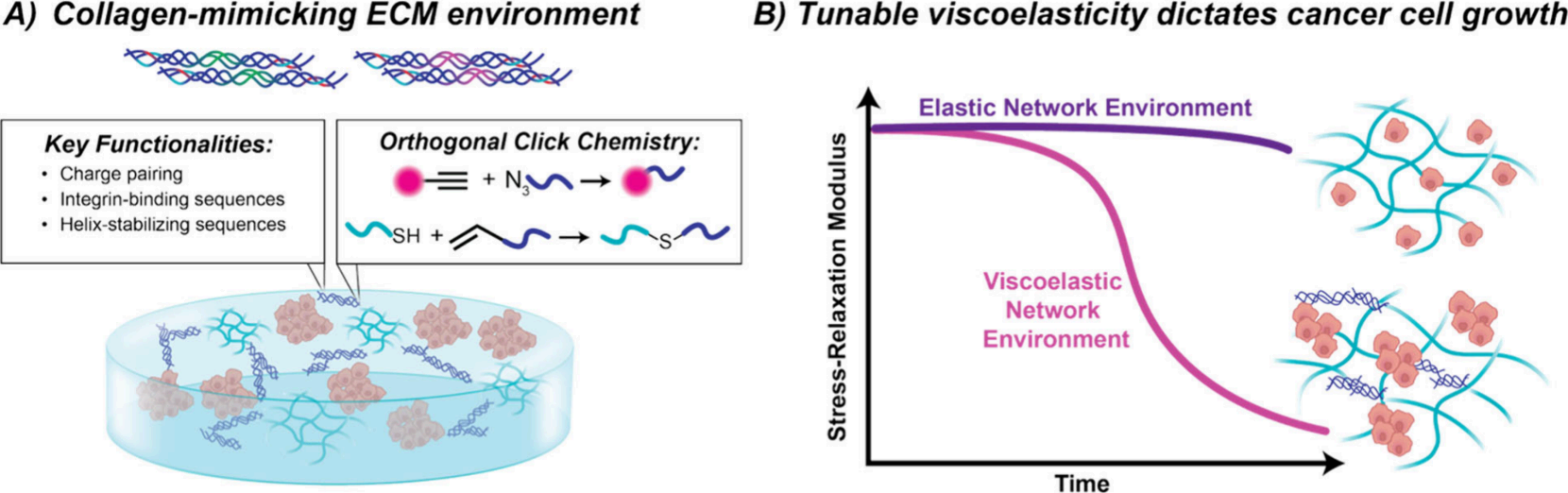
(A) Hydrogels prepared by cross-linking collagen-mimicking peptides (CMPs) with four-arm PEG-thiol create a bioactive ECM-like 3D environment for studying breast cancer cell growth. (B) Tunable relaxation times in bioactive CMPs, as influenced by variable extents of physical cross-links.

Building on the fundamental importance of soft structures in biology, the authors successfully replicate essential mechanical features of the extracellular matrix (ECM) in a fully synthetic, abiotic system.

The authors’ use of chemical synthesis and orthogonal click reactions underpins key advances and reinforces the power of thiol–ene and azide–alkyne coupling in materials chemistry, both examples of reactions with extraordinary influence across soft materials science and the bio- and cell-polymer interface.^6–8^ Their integration of alloxycarbonyl groups (i.e., alloc, normally a protecting group but here a thiol–ene coupling precursor) and azide functionality into collagen-mimicking peptides (CMPs) prepares these structures for hydrogel formation and labeling with Alexa Fluor dyes, enabling systematic examination of structure, mechanics, and cell response. Readers will note the prominent role of efficient peptide synthesis: integration of well-defined oligopeptide sequences (“blocks”) stabilizes triple helix macromolecular peptide self-assembly, promotes binding, and drives fibril formation. However, even a low level of coupling errors produces quantifiable amounts of difficult-to-remove impurities, which the authors convey as an ongoing challenge when peptide synthesis involves dozens or more coupling steps and includes unconventional amino acids.

The authors complemented their characterization of fibrillar helicity using circular dichroism with advanced fluorescence spectroscopy techniques that enable visualization of structure and morphology. Indeed, super-resolution optical reconstruction microscopy (STORM) images in Figure 3 of ref 1, produced by stochastic blinking of the covalently attached Alexa Fluor dyes, highlight the fibrillar nature of these polypeptide assemblies at sub-50 nm resolution. Such visualization of hydrogel-embedded fibrillar structures further demonstrates the power of click conjugation and gives access to microscopic characterization that obviates the need for electron microscopy techniques that would be cumbersome on hydrophilic gels of this type. Indeed, their use of STORM offers an instructive example for researchers seeking to adapt imaging techniques that originated in the neuro- and cell biology sciences^9^ for deepening understanding of synthetic, biomimetic materials.

Their use of STORM offers an instructive example for researchers seeking to adapt imaging techniques that originated in the neuro- and cell biology sciences for deepening understanding of synthetic, biomimetic materials.

Particularly striking is the authors’ description of the interplay among hydrogel composition, relaxation time, and cell response, as they find that viscous dissipation of the networks is enhanced by embedding functional, collagen-mimicking structures. Taking inspiration from the sliding action of assembled collagen I units in native tissue, the authors conceptualized and produced CMP-based networks with “collagen-like” physical cross-links that promote stress dissipation with applied strain. The effect is amplified further by increasing the concentration of these multifunctional peptides within the hydrogel. Figure 4b in ref 1 reinforces how this approach allows precise chemical control over the rheology of these gels, where the (predominantly soft/elastic solid) material becomes increasingly dissipative and more fluid-like at higher peptide concentrations—reducing the relaxation time by 2–3 orders of magnitude and emulating the mechanical behavior of clinically relevant viscoelastic scaffolds.

Maintaining the rheological properties in hydrogels that also contain integrin-binding peptides deepens their relevance for cell encapsulation and modulation of cell response, which is critically important for understanding cancer cell types known to metastasize more easily in more dissipative surroundings. Indeed, the authors note that 70% of newly diagnosed breast cancers are sensitive to the mechanical properties of their environment. Mechanisms are proposed to connect different microstructural self-assemblies at different concentrations (triple helix vs fibrillar assembly) to the rheological response across concentrations and length scales. As shown in Figure 5b of ref 1, the authors cultured T47D breast cancer cells over 7 days in these novel peptide-containing gels and demonstrated a statistically significant difference in average cluster volume for different chemical compositions of the gels (corresponding to tuning only of the mechanical response rather than bioactivity). Extensive details in their cell proliferation studies illustrate the importance of decoupling the role of viscoelasticity from bioactivity.

Maintaining the rheological properties in hydrogels that also contain integrin-binding peptides deepens their relevance for cell encapsulation and modulation of cell response, which is critically important for understanding cancer cell types known to metastasize more easily in more dissipative surroundings.

The importance of Kloxin and co-workers’ paper cannot be overstated, as their approach to bioactive, mechanically tunable materials sets up the broader community to build in new directions on all fronts—chemical, mechanical, and biological—toward fundamental understanding of the complex underlying mechanisms of cell proliferation, differentiation, transduction, and related events. Their sophisticated integration of chemistry, mechanics, and biology serves as a powerful example of forward-looking materials science that hinges on in-depth interdisciplinary cooperation so as to enable exciting new discoveries.
